# Exploring E-Health Literacy and Technology-Use Anxiety among Older Adults in Korea

**DOI:** 10.3390/healthcare11111556

**Published:** 2023-05-25

**Authors:** Jiyoun Kim, Sang-Wan Jeon, Hyun Byun, Eunsurk Yi

**Affiliations:** Department of Exercise Rehabilitation, Gachon University, 191 Hambakmoe-ro, Yeonsu-gu, Incheon 21936, Republic of Korea

**Keywords:** middle-aged and older adults, e-health literacy, technology-use anxiety, latent mean analysis

## Abstract

The COVID-19 pandemic has increased the importance of health literacy in disseminating information on health in a non-contact society. This study focused on examining the acceptance capacity by older adults of smart devices in Korea and investigating the potential differences between men and women in terms of e-health literacy and technology-use anxiety. The study included 1369 respondents who were adults over 50 years of age and used welfare centers, public health centers, senior citizen centers, and exercise centers in Seoul and Incheon. An online survey was conducted from 1 June 2021 to 24 June 2021. The study found that the older adults’ low levels of digital literacy could limit their access to health information and negatively impact their health. The difference between men and women in terms of technology-use anxiety was statistically significant, with the latent mean for men being higher than that for women. The effect sizes of the potential mean differences were found to be at a medium level for e-health literacy and a significant level for technology-use anxiety. With Korea’s aging population and the need for the continuous management of chronic diseases among older adults, it is essential to discuss internet-based health information for disease maintenance and treatment.

## 1. Introduction

As the non-contact society expands due to the COVID-19 pandemic, a new concept of health literacy is becoming essential in the current medical system for the correct dissemination of information on digital health [1]. In the current global COVID-19 crisis, the development of vaccines and treatments is still in progress, and maintaining social distance remains one of the most effective solutions to containing the pandemic [2]. This underscores the role and importance of interventions based on digital health technology. In the information age, personal health management has become easier due to the availability of internet-based health information using digital devices to enable self-diagnosis [3]. As the amount of health information provided online increases, the internet will increasingly become an essential source of health information [4,5]. In the United States, approximately six out of ten adults search for health information online [3].

In Korea, as older adults’ internet use increases due to the general accessibility of the mobile internet environment [6], the need to manage and prevent chronic diseases in older adults by using a health management program based on mobile technology is becoming more critical. Korea is currently expanding mobile healthcare services through public health centers to provide information on lifestyle improvement, chronic disease prevention, and management services to more chronic disease risk groups [7]. The increase in the use of information and communication technologies in health-related fields not only provides opportunities to improve and promote health but also raises additional concerns about solving new problems.

Health literacy refers to how individuals acquire, process, understand, and communicate health information and is known to be correlated with individuals’ health statuses [8]. As the production and distribution of information rapidly expand and their impacts on life increase, low levels of health literacy can negatively affect not only individual health behaviors but also health management [9]; moreover, the lower people’s levels of health literacy, the more negatively they perceive their health statuses [10] and the higher the associated medical expenses due to more frequent hospital visits [11,12]. In particular, e-health literacy can be a factor that reflects an individual’s ability to understand and interpret health-related data on the internet, and as a result, it can help individuals achieve better health outcomes [13].

A survey which comprehensively measured the availability of internet access, PC and mobile device usage, and internet utilization as they relate to the digital information gap in Korea has shown that the digital literacy level for four vulnerable groups (disabled people, low-income groups, farmers and fishermen, and older adults) was 72.7% compared to the general population. Although it had improved by 2.8% compared to the previous year (69.9%), the digital literacy level of older adults was 68.6% compared to the general public [14]. In other words, the low levels of digital literacy for older adults may cause problems in their ability to obtain and understand health information, which may negatively affect their health. In particular, as Korea’s population ages and as most chronic disease patterns of older adults require continuous management, it is essential to discuss alternatives in the pursuit of internet health information for the maintenance of the health and treatment of diseases in older adults.

Health literacy is strongly correlated with socioeconomic factors such as level of education [15,16]. If we look at the literature on older adults’ ability to access internet health information and their internet health comprehension, much of the existing research has focused on the concept, definition, and relevance of health literacy [17]. These studies were conducted on an individual level and on the level of organizations, communities, and healthcare systems [18]. Research on the socioeconomically vulnerable is conducted continuously so that the gap in health literacy does not lead to health inequality; however, gender differences relating to internet health information are not sufficiently discussed [19].

Since it is necessary to understand individual users’ points of view regarding the diffusion of new technologies [20], it is necessary to pay attention to users’ technology acceptance prior to their behavior patterns in terms of technology use. A technology acceptance attitude refers to an individual’s positive or negative attitude toward a specific technology [21]. Technology-use anxiety [22] can negatively impact the use of technology and attitudes toward technology acceptance when targeting older adults [22,23,24]. If the problem of technology-use anxiety among older adults is not addressed, discriminatory results may appear in public health due to older adults’ technology use and skill gaps. Therefore, it is necessary to study ways to alleviate the harmful effects of technology-use anxiety among older adults concerning new technologies.

It is also important to understand the degree of e-health literacy and anxiety related to technology use among older Koreans by gender and to participate in mobile and internet health interventions because the prevalence of chronic diseases is increasing. In addition, the digital information gap is increasing, despite the increase in the use of digital devices; moreover, the intention to manage health using digital devices is also increasing. From this perspective, an analysis of older adults’ smart device capacities can be used as primary data to help them manage their health using digital technology. Research findings indicate a gender disparity in digital literacy, with women facing more challenges and exhibiting lower levels of confidence in using technology compared to men [25]. Hence, interventions to enhance digital literacy among older adults should prioritize addressing these gender differences. Additionally, while existing studies have generally reported lower levels of digital literacy among older individuals with lower levels of education and lower incomes, as well among those of advanced age, the COVID-19 pandemic has had a widespread impact on the older adult population, regardless of their socio-economic backgrounds [26]. Consequently, as society transitions to a rapidly evolving non-face-to-face environment, all older adults, irrespective of their geographical locations, education levels, or incomes, have experienced a sense of powerlessness [27]. This situation emphasizes the urgent need to achieve universal digital welfare. Furthermore, investigating the digital skills of older adults who are relatively privileged and educated could provide insights into providing digital welfare services to older adults in general. Notably, the differences in digital literacy observed among highly educated older adult groups can serve as compelling evidence to support the necessity of universal digital welfare in the context of the COVID-19 pandemic. This study differs from previous studies in that it supplements other empirical studies by focusing on the characteristics of older adults. In addition, analyzing the degree of e-health literacy and technology-use anxiety by gender will make it possible to tailor education policies related to internet health information education based on the characteristics of older adults.

This study first verified the construct equivalence (i.e., form identity, measurement identity, and intercept identity) through a multi-group confirmatory factor analysis of the smart device acceptance capacity scale for older adults in Korea, and then it examined whether this study could commonly apply the acceptance capacity scale. Secondly, a latent mean analysis was used to verify potential gender differences between older adults in Korea for two factors (e-health literacy and technology-use anxiety) in terms of their smart device acceptance capacities. Consequently, the below research questions were proposed.

First, how is the construct equivalence (i.e., form, measurement, and intercept equivalence) verified according to the smart device capacity scale for older Korean adults?

Second, what are the potential differences in smart device acceptance capacities (e-health literacy and technology-use anxiety) based on the gender of older adults in Korea?

## 2. Research Method

### 2.1. Research Subjects

The subjects of this study were Korean adults over 50 who used welfare centers, public health centers, and senior citizen centers in Seoul and Incheon. The survey was conducted online from 1 June 2021 to 24 June 2021. The survey produced 1500 completed questionnaires. The data from 1369 people were used as the sample after excluding 131 respondents because their answers were either insincere or their questionnaires were incomplete or indicated low reliability. Erdfelder and Buchner [28] used a priori power analysis to determine the appropriate sample size above the minimum number required to interpret a study’s results. They suggested that the required sample size should be 210 or more when the effect size (d) is set to 0.50, the α level is 0.05, and statistical power is set at 0.95. In this study, as a result the calculations performed by G*POWER 3.0, the required sample size was 210 people (d = 0.50, α = 0.05, and 1 − β = 0.95). The general characteristics of the study’s subjects are summarized in Table 1.

### 2.2. Measuring Tool

In this study, a questionnaire was used as the data collection tool to analyze the latent mean of the e-health literacy and technology-use anxiety scales according to the gender of older adults living in Korea. The questionnaire was used after modifying the measurement tools of the variables used in previous studies. For e-health literacy, eight items on the e-health literacy scale (eHEALS) developed by Norman and Skinner [29] were modified and supplemented with terms and sentences suitable for this study. In the case of technology-use anxiety, four computer anxiety questions used in the study by VenKatech and Bala [30] were modified and supplemented with terms and sentences suitable for technology-use anxiety in smart devices. The maximum likelihood method with a direct Oblimin rotation was used for the exploratory factor analysis of the validity test. The reliability test was performed using Cronbach’s alpha to check for internal consistency. As a result of the exploratory factor analysis, one item of technology-use anxiety showed a factor load of 4.0 or less. After deleting the item, the exploratory factor analysis was repeated to confirm the validity of the factors for e-health literacy and technology-use anxiety [31]. The re-analysis found that the variance occupied by the eleven questions (eight questions for e-health literacy and three questions for technology-use anxiety) was 78.17%. In addition, the load was 0.40 or higher for all the questions, and the internal consistency ratings between the questions for each selected factor were 0.957 for e-health literacy and 0.876 for technology-use anxiety, indicating relatively high reliability. The final questionnaire consisted of eight questions for e-health literacy, three questions for technology-use anxiety, and five questions for demographic characteristics, and it was used as a measurement tool in this study. In addition, the factors related to e-health literacy and technology-use anxiety were named and used as indicators of smart device capacity. The compositions of the questionnaires are summarized in Table 2.

### 2.3. Data Processing

AMOS 22.0 was used in the analysis of e-health literacy and technology-use anxiety based on the gender of older adults living in Korea. To analyze the latent mean, the morphological identity, measurement identity, and intercept identity were verified through a confirmatory factor analysis of each measure, and then the factor variance identity was verified. The effect size was calculated while performing the latent mean analysis. To confirm the general characteristics of the respondents, descriptive statistics were performed using SPSS/WIN 22.0. Exploratory factor and reliability analyses using Cronbach’s alpha were performed to establish the validity and reliability of the survey tool (Table 3). Moreover, the suitability of the data for the structural equation model was verified through descriptive statistics and a correlation analysis of the items.

## 3. Results

### 3.1. Suitability of the Material

To verify the suitability of the data, descriptive statistics such as means, standard deviations, kurtosis, skewness, and correlation analysis were utilized. The results are summarized in Table 4. First, the normalities of the e-health literacy and technology-use anxiety scales were checked as the criteria for kurtosis and skewness. In general, although scholars have differed on the standard for data normality, the conditions for a normal distribution in the structural equation model proposed by Hong [32] are satisfied when the skewness is less than 2.0 and the kurtosis is less than 4.0. Thus, we confirmed that the measurement items for each sub-item of the e-health literacy and technology-use anxiety scales also met this standard. In addition, we were able to confirm that there was no problem with multicollinearity because the correlation between the variables was lower than the standard value multicollinearity of 0.80.

### 3.2. Structural Model (Basal Model) Analysis

**Figure 1:**
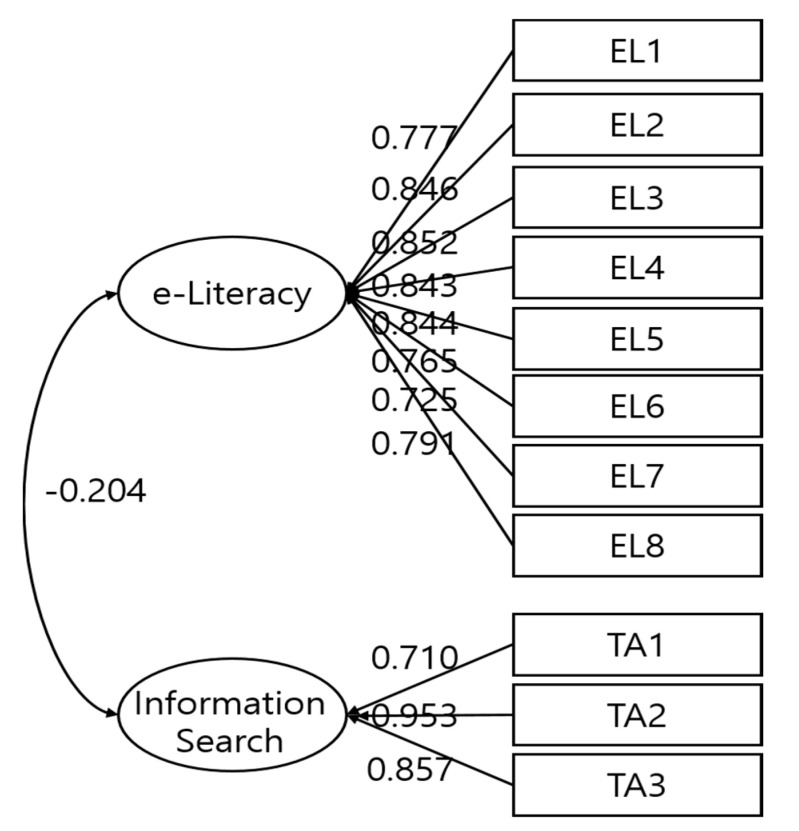
Structural model path coefficient.

The analysis of the structural models of the e-health literacy and technology-use anxiety scales for older adults living in Korea (Table 5) confirmed that all the fitness levels were satisfied with standard values. The relationship structure between the measured variables was consistent with the empirical data. It is desirable to evaluate the goodness-of-fit of a model with a goodness-of-fit index that is not sensitive to the sample size, considers the model’s simplicity at the same time, and has established clear interpretation criteria [33]. If the fitness indices that satisfy these criteria are the comparative fit index (CFI), Tucker–Lewis index (TLI), and root mean square error of approximation (RMSEA), then it can be considered a relatively good fitness index if the TLI and CFI are 0.90 or more and the RMSEA is 0.08 or less [34]. In this study, x^2^ was 536.519 (df: 43.00, *p* < 0.05), the CFI was 0.975, the TLI was 0.965, and the RMSEA was 0.077, indicating a good model-fit index. Based on these results, the structural model was determined to be a suitable model for the latent mean analysis. The relationship between variables can also be interpreted through the estimated path coefficients (Figure 1). After confirming that there was no problem in the structural model through the conformity assessment of the structural model, a construct equivalence verification (morphological equivalence, measurement equivalence, and intercept equivalence) was performed. The factor variance equivalence was verified, and the latent mean analysis was performed. The results are summarized in Table 5.

### 3.3. Latent Mean Analysis by Construct Equivalence Verification

In order to verify the morphological identity of the e-health literacy and technology-use anxiety scales for older adults living in Korea, the measurement models for each factor were compared with those of the men and women in the sample (Table 6). Conformational invariance is a measure of fit that evaluates whether the measurement models of the groups being compared are the same. In this study, the fit index of conformational identity allowed for correlation by factor, and it also allowed parameter values to be freely estimated. A goodness-of-fit (x^2^ = 469.139, TLI = 0.961, CFI = 0.966, and RMSEA = 0.053) was obtained, and conformational identity was satisfied.

Next, the values for the morphological identity (Model 1) and measurement identity (Model 2) and the degrees of freedom (df) were compared in the measurement model with the same constraint on the same factor loading. Metric invariance verifies whether the measurement variables for each group are at the same level. The fit was compared with the model for which the morphological uniformity was verified. Since the morphological identity is a model inherent in measurement identity, it was verified through the difference in values using the difference in the degrees of freedom (df) between the two models. Because it was found to be statistically insignificant ($\Delta x^{2}$(2, n = 1388) = 43.965, *p* > 0.05), the e-health literacy and technology-use anxiety scales were the same for both the men and the women. Since the measurement identity was established, the intercept identity (Model 3) was confirmed. Scalar invariance, which verifies whether the intercept of each measurement variable is the same between groups, was verified by comparing the fit with the model for which the measurement equality was verified. In this study, there was no statistically significant difference in the values of the fit index between the measure identity and the intercept identity of each measured variable with the same restrictions ($\Delta x^{2}$(2, n = 1388) = 151.909, *p* > 0.05). In addition, the fitness indices (the TLI, CFI, and RMSEA) satisfied the fitness criteria ($\Delta x^{2}$(101, *p* < 0.5) = 665.013, TLI = 0.949, CFI = 0.953, RMSEA = 0.037).

Kim [35] noted that the equality constraint is not rejected if the index of the model to which the intercept equality constraint is applied is not significantly worse than the index of the model to which the measurement equality constraint is applied. Therefore, intercept homogeneity was established because the difference between the fitness indices for the measurement and intercept uniformity was insignificant in the measurement models of the e-health literacy and technology-use anxiety scales.

Combining these results, the primary conditions for verifying whether the measurement variables could be used for older adults according to gender were satisfied, and the intercept of the measurement tool was applied in the same way. These results can be interpreted as reflecting the actual difference between the groups for the latent factors.

Next, factor variance equality was verified by construct equivalence satisfaction. Factor variance identity is used to compare the fit with the model whose intercept identity has been verified through the constraint of equality on the variance of the latent factors of the group. These processes cannot be simultaneously verified and should be identified through a hierarchical stage. If there are no problems, a latent means analysis using latent factors with controlled measurement errors should be performed to measure the differences between groups [31]. In addition, to present an index that meets the measurement criteria, it is necessary to calculate the effect size, which is calculated by applying a typical standard deviation when the typical variances of the latent factors calculated from both groups are the same. As a result of first analyzing the identity of factor variance through this process (Table 7), the fit index showed little change with the intercept identity, confirming that the identity of the factor variance was secured. When presenting Cohen’s effect size (d) together with the difference in the latent mean, the factor variance equality must be verified because the typical standard deviation is applied when the variances of the latent factors calculated from the two groups are the same. In this study, there was no problem even when the typical standard deviation was used.

We then analyzed the latent mean and effect size. The results are summarized in Table 8. Since a latent mean analysis cannot directly conduct estimations, the latent mean of a comparison group is assumed to be zero, and then the latent mean of the measurement group can be measured and compared [36]. In latent mean analysis, a constant of one is used as an independent variable for each factor. The regression coefficient estimated here becomes the mean of the factors, that is, the latent mean [37]. Therefore, to estimate the latent mean, the latent male average was set to zero. We then checked whether there was a difference In the older adults. To check the significance of the difference in the calculated latent mean, a value that meets the measurement criteria is presented [31,38] and compared with the effect size of Cohen [39]. When looking at the effect size criterion in the criteria presented by Cohen [39], if the value of the effect size (d) is less than 0.20, it is interpreted as a small level; if it is 0.50, it is interpreted as a medium level; and if it is above 0.80, it is interpreted as a large level. In this study, the effect size of the e-health literacy (0.67) factor exceeded the median level of 0.50, and so the effect size of a medium level was present, and thus, there was anxiety about the use of smart device technology (1.15). The factors were found to have a significant effect size. As shown in Table 8, there was no significant gender difference in the e-health literacy scale (−0.015) by the analysis of variance and the latent mean analysis. However, there was a significant gender difference in the technology-use anxiety scale (0.138, *p* < 0.05).

## 4. Discussion

This study conducted a latent mean analysis of e-health literacy and technology-use anxiety in older Korean men and women. To establish whether e-health literacy and technology-use anxiety are similar across the genders in older adults living in Korea, we conducted a hierarchical analysis of the morphological identity, measurement identity, and intercept identity. The results confirmed that e-health literacy and technology-use anxiety were common among older Korean men and women, and that the observed differences in the means reflected the actual differences between the groups for the latent factors. Until now, the *t*-test and analysis of variance—the usual methods for comparing mean differences between groups—have had decisive weaknesses in that they do not take into account measurement errors [40,41]. The latent mean analysis method provides a more valid study result because it tests the mean difference using a latent variable with a controlled measurement error, and this suggests that the procedure used in this study was appropriate.

The latent mean analysis of e-health literacy and technology-use anxiety in this study showed a statistically significant difference between the genders only for technology-use anxiety. In addition, the potential average for the men was slightly higher. Based on Cohen’s effect size, e-health literacy showed a medium effectiveness of 0.67 and technology-use anxiety was at a level of 1.15.

The latent mean analysis has certain limitations, as it is a methodology that can only be executed when the assumptions of configuration invariance, metric invariance, and scalar invariance are met through multi-group confirmatory factor analysis. A limitation of the latent model is that it can solely accommodate MTMM (multitrait-multimethod) data if the indicators are completely homogeneous. This means that all indicators within each method have entirely correlated true score variables, which vary solely in scaling, implying they may have different intercepts and loadings [39]. Furthermore, if the latent model presumes that the correlation structure is homogeneous across the methods for a given construct, it may not fit the data accurately. To overcome these challenges, Eid [40] proposed a solution that involves incorporating indicator-specific residual factors for all indicators except for a reference one.

Previous studies have generally divided the factors influencing e-health literacy into personal, situational, and environmental factors [42], and they have shown that demographic and sociological characteristics also have an influence [43]. Studies conducted in Europe and Taiwan have found that men report lower levels of health literacy than women [44,45]. The higher the social status, education level, and economic level, the higher the found level of e-health literacy [46,47,48]. This study found no statistically significant difference in e-health literacy between men and women, indicating that gender does not directly affect e-health literacy, as previous studies have suggested. In addition, there was a slight difference compared to the results of previous studies in that the men had a higher understanding of internet health information than the women when looking at the average values. Older adults, referred to as the baby-boomer generation in Korea, represent a generation that is economically, socially, and politically dominant, and they are an economically stable age group. In particular, the patriarchal concept, which is characteristic of this generation, is based on men’s economic superiority. The results of previous studies that showed a correlation between economic status and internet health information comprehension were partially supported by the results of this study.

In general, physiological and psychological functions change as aging progresses. This change also affects technology-use anxiety and appears as a heterogeneous response among older adults, in particular [21], because user perception of the helpfulness and user-friendliness of technology can be regarded as an essential factor in using technology [49]. A study that analyzed differences in mobile health app use found that gender was not associated with general use, but women were more likely to use nutrition, self-care, and reproductive health apps [50]. In other words, while is difficult to conclude that there is a specific difference in the use of internet health information according to gender, there are some differences in the type of health information obtained. As for technology acceptance anxiety, this study’s finding that men experience more significant anxiety indirectly supports the results of previous studies that showed that older men (66 years and older) were less health literate than women [51]. Sun and Zhang [52] found that men’s acceptance or intention to use technology was more positive because men were more likely than women to engage in new technology or device used as a means to achieve a specific goal. Their finding differs from the results of this study, but a careful interpretation is required because the age groups of the subjects differed. However, it is necessary to consider the possibility of various interpretations and to understand the differences in the results of smart device capacity according to gender in terms of environmental context.

According to Lawton and Nahemow [53], humans experience the aging process while adapting to a changing environmental context; moreover, the aging experience depends on context [54]. In other words, even if an older adult feels anxious about using new technologies on a personal level, if an environment that compensates for such anxiety is provided, the negative effect of technology-use anxiety will decrease. Technology-use anxiety will be reduced by an older adult’s acceptance of technology. Therefore, for older adults who are reluctant to use smart device technology, the e-health literacy strategy should be adjusted based on their educational and digital literacy levels [45].

This study has a number of limitations. First, this study’s questionnaire data were collected through an online survey. The possibility that older adults who were relatively accustomed to using computers and the internet would have participated in the survey cannot be excluded. In addition, considering the descriptive statistics of the study’s participants, it is highly likely that they represented older adults, and their education and income levels tended to be relatively higher than any other age group in Korea. In addition, this study assumed that older adults are a homogeneous group. In addition, this study only focused on e-health literacy, technology-use anxiety, and demographic characteristics as factors in smart device capacity.

Unlike earlier studies, this study focused on analyzing the differences between e-health literacy and technology acceptance anxiety. By using gender, which is a demographic characteristic, as a representative variable, and by analyzing gender characteristics in greater depth, future studies could provide information that could help to identify characteristics related to e-health literacy and technology acceptance anxiety in greater detail.

The results of this study showed that there was no statistically significant difference in the smart device capacity between older men and women living in Korea. However, there was a significant difference that depended on intentions and behavioral characteristics. The significant differences between men and women regarding the technology acceptance anxiety related to smart devices may cause a health gap in the future. It is, therefore, necessary to identify and remove obstacles that may affect the use of smart devices based on gender characteristics and to lower psychological anxiety about technology acceptance.

## 5. Conclusions

This study conducted an analysis of construct equivalence and latent mean according to gender in a smart device capacity scale for older adults living in Korea. The below conclusions were obtained.

First, as a result of a multi-group confirmatory factor analysis of the smart device acceptance capacity scale of older adults living in Korea, the construct equivalence was verified because the morphological equivalence, measurement equivalence, and intercept equivalence were all satisfied. It was possible to compare the differences in the latent factors by group as each latent variable and measurement variable for the men and women were equally applied to e-health literacy and technology-use anxiety.

Second, as a result of the latent mean analysis, the average difference between the groups regarding technology-use anxiety as a factor in smart device acceptance capacity was statistically significant, and the latent average for men was higher than that for women. When looking at the effect size of the potential mean difference, e-health literacy was 0.67 and technology-use anxiety was 1.15, showing a medium and a large effect size, respectively, based on the criteria suggested by Cohen. In follow-up research, it will be necessary to continuously check and revise the validity of the various variables that affect the acceptance of smart devices and develop and apply a new factor structure based on the results.

Lastly, one limitation of this study was the potential bias in the sample used for the research. The study included respondents who were adults over 50 years of age that used welfare centers, public health centers, senior citizen centers, and exercise centers in Seoul and Incheon, which may not be representative of the broader population of older adult individuals in Korea. Furthermore, the sample may have been biased towards those who were digitally literate and comfortable using technology, potentially limiting the generalizability of the study’s findings to a broader population of older adult individuals who may not be as technologically proficient. While the study’s findings provided valuable insights into the potential differences in e-health literacy and technology-use anxiety between men and women, it is essential to recognize the limitations of the sample used and interpret the study’s results with caution. Future research could adopt more inclusive sampling methods to ensure that the perspectives of a more diverse group of older adult individuals are represented.

## Figures and Tables

**Table 1:** General characteristics of the study’s subjects.

Division	Contents	Number of Respondents	Frequency (%)
Gender	Men	770	56.2
	Women	599	43.8
Age	50–54	417	30.5
	55–59	254	18.6
	60–64	444	32.3
	Older than 65	254	18.6
Education	Lower than elementary school	12	0.8
	Middle school	39	2.8
	High school	348	25.6
	University graduate or higher	970	70.8
Exercise frequency	Not at all	391	28.6
	Once per week	216	15.8
	Twice per week	226	16.5
	3 times per week	209	15.3
	4 times per week	89	6.5
	5 times per week	111	8.0
	6 or more times per week	127	9.3
Economic status	Very economical	11	0.8
	Able to afford	161	11.8
	Average	826	60.3
	Financially difficult	320	23.4
	Very difficult financially	51	3.7

**Table 2:** Compositions of the questionnaire.

Dimensions	Measuring Items	Question Number
**e-health literacy**		Norman and Skinner [26]
EL1	I know health-related resources are accessible through smart devices.	
EL2	I know where to find useful health-related resources on smart devices.	
EL3	I know how to find useful health-related resources on smart devices.	
EL4	I know how to use smart devices to find answers to health-related questions.	
EL5	I know how to utilize health-related information.	
EL6	I have the skills necessary to evaluate health-related information.	
EL7	I can distinguish between low-quality and high-quality health-related resources.	
EL8	I am confident in using information from smart devices for health-related decisions.	
**Technology-use anxiety**		VenKatech and Bala [27]
TA1	I am afraid of using smart devices.	
TA2	I do not want to be bothered with using a smart device.	
TA3	I am uncomfortable using smart devices.	
TA 4	I am nervous using smart devices.	

**Table 3:** Exploratory factor analysis of the e-health literacy and technology-use anxiety scales.

	Smart Device Capacity		
	e-Health Literacy	Technology-Use Anxiety	Cronbach’s Alpha
EL1	0.822	−0.236	0.957
EL2	0.874	−0.210	
EL3	0.883	−0.210	
EL4	0.878	−0.220	
EL5	0.868	−0.230	
EL6	0.802	−0.118	
EL7	0.750	−0.101	
EL8	0.810	−0.220	
TA1	−0.217	0.708	0.876
TA2	−0.183	0.962	
TA3	−0.170	0.855	
Eigenvalues	6.439	2.161	
Dispersion %	58.534	19.645	
Cumulative variance %	58.534	78.179	

Note: EL, e-health literacy; TA, technology-use anxiety.

**Table 4:** Correlation between the variables and the descriptive statistics.

	Smart Device Capacity										
	e-Health Literacy								Technology-Use Anxiety		
	EL1	EL2	EL3	EL4	EL5	EL6	EL7	EL8	TA1	TA2	TA3
EL1	1										
EL2	0.753 **	1									
EL3	0.689 **	0.791 **	1								
EL4	0.635 **	0.692 **	0.718 **	1							
EL5	0.625 **	0.683 **	0.708 **	0.781 **	1						
EL6	0.553 **	0.606 **	0.606 **	0.620 **	0.654 **	1					
EL7	0.498 **	0.553 **	0.575 **	0.599 **	0.600 **	0.694 **	1				
EL8	0.590 **	0.628 **	0.640 **	0.645 **	0.660 **	0.698 **	0.700 **	1			
TA1	0.471 **	0.460 **	0.453 **	0.469 **	0.458 **	0.396 **	0.387 **	0.508 **	1		
TA2	−0.174 **	−0.152 **	−0.155 **	−0.205 **	−0.175 **	−0.151 **	−0.135 **	−0.208 **	−0.339 **	1	
TA3	−0.236 **	−0.217 **	−0.207 **	−0.232 **	−0.211 **	−0.124 **	−0.103 **	−0.205 **	−0.392 **	0.677 **	1
Mean	30.47	30.43	30.46	30.51	30.43	30.12	30.23	30.24	30.70	30.12	20.92
Standard deviation	0.719	0.765	0.795	0.837	0.855	0.888	0.852	0.919	10.364	10.332	10.328
Skewness	−0.406	−0.371	−0.300	−0.467	−0.485	−0.237	−0.276	−0.265	−0.162	0.344	0.527
Kurtosis	0.133	−0.099	−0.164	−0.038	−0.008	0.024	0.226	−0.095	−0.656	−0.444	−0.179

**, *p* < 0.01

**Table 5:** Structural model (basal model) fit analysis.

Index Name	Exponent Value	Criterion Value	Fitness
x^2^(df,p)	536.519 (43.00)	>0.5	Fit
CFI	0.975	≤0.90	Fit
TLI	0.965	≤0.90	Fit
RMSEA	0.077	≥0.08	Fit

Note: CFI, comparative fit index; TLI, Tucker–Lewis index; RMSEA, root mean square error of ap- proximation.

**Table 6:** Goodness-of-fit index for identity verification.

	x^2^	df	TLI	CFI	RMSEA
Model 1	469.139	97	0.961	0.966	0.053
Model 2	513.104	99	0.962	0.965	0.055
Model 3	665.013	101	0.949	0.953	0.063

**Table 7:** Factor variance equality test.

	x^2^	df	TLI	RMSEA
Model 3	686.135	102	0.953	0.063
Model 4	693.559	113	0.957	0.060

Note: Model 3, scalar invariance; Model 4, factor variance invariance.

**Table 8:** Analysis of the differences in the latent means between the men and women for the variables.

		Male (n = 806)		Female (n = 631)		d	TM
		LMA	M	LMA	M		
Smart device capacity	e-health literacy	0	2.91	−0.015	2.88	0.67	2.89
	Technology-use anxiety	0	3.69	0.138 *	3.68	1.15	3.68

Note: *, *p* < 0.05; d, effect size; LMA, latent mean analysis; M, mean; TM, total mean.

## Data Availability

The data are available upon reasonable request to the corresponding author.

## References

[1] Sentell T., Vamos S., Okan O. (2020). Interdisciplinary perspectives on health literacy research around the world: More important than ever in a time of COVID-19. Int. J. Environ. Res. Public Health.

[2] World Health Organization (2020). COVID-19 and Digital Health: What Can Digital Health Offer for COVID-19.

[3] Fox S., Duggan M. (2013). Health Online 2013. Information Triage.

[4] Fox S. (2007). E-patients with a Disability or Chronic Disease.

[5] Pang P.C.I., Chang S., Verspoor K., Pearce J. (2016). Designing health websites based on users’ web-based information-seeking behaviors: A mixed-method observational study. J. Med. Internet Res..

[6] National Information Society Agency (2021). 2020 Survey on the Internet Usage.

[7] Ministry of Health and Welfare (2018). Public Health Center Mobile Healthcare. https://www.mohw.go.kr/react/al/sal0301vw.jsp?PAR_MENU_ID=04&MENU_ID=0403&CONT_SEQ=345280/.

[8] Berkman N.D., Davis T.C., McCormack L. (2010). Health literacy: What is it?. J. Health Commun..

[9] DeMarco J., Nystrom M. (2010). The importance of health literacy in patient education. J. Consum. Health Int..

[10] Vozikis A., Drivas K., Milioris K. (2014). Health literacy among university students in Greece: Determinants and association with self-perceived health, health behaviours and health risks. Arch. Public Health.

[11] Weiss B.D., Palmer R. (2004). Relationship between health care costs and very low literacy skills in a medically needy and indigent Medicaid population. J. Am. Board Fam. Pract..

[12] Baker D.W., Wolf M.S., Feinglass J., Thompson J.A., Gazmararian J.A., Huang J. (2007). Health literacy and mortality among elderly persons. Arch. Intern. Med..

[13] Zhou J., Fan T. (2019). Understanding the factors influencing patient E-health literacy in online health communities (OHCs): A social cognitive theory perspective. Int. J. Environ. Res. Public Health.

[14] National Information Society Agency (2021). 2020 The Report on the Digital Divide.

[15] Beauchamp A., Buchbinder R., Dodson S., Batterham R.W., Elsworth G.R., McPhee C., Sparkes L., Hawkins M. (2015). Distribution of health literacy strengths and weaknesses across socio-demographic groups: A cross-sectional survey using the Health Literacy Questionnaire (HLQ). BMC Public Health.

[16] HLS-EU Consortium (2012). Comparative Report of Health Literacy in Eight EU Member States: The European Health Literacy Survey HLS-EU. https://cdn1.sph.harvard.edu/wp-content/uploads/sites/135/2015/09/neu_rev_hls-eu_report_2015_05_13_lit.pdf/.

[17] Sørensen K., Van den Broucke S., Fullam J., Doyle G., Pelikan J., Slonska Z., Brand H. (2012). Health literacy and public health: A systematic review and integration of definitions and models. BMC Public Health.

[18] Peerson A., Saunders M. (2009). Health literacy revisited: What do we mean and why does it matter?. Health Promot. Int..

[19] Oliffe J.L., Rossnagel E., Kelly M.T., Bottorff J.L., Seaton C., Darroch F. (2020). Men’s health literacy: A review and recommendations. Health Promot. Int..

[20] Jung M.M., Ludden G.D. (2019). What do older adults and clinicians think about traditional mobility aids and exoskeleton technology?. ACM Trans. Hum.-Robot Int..

[21] Chen K., Chan A.H.S. (2014). Gerontechnology acceptance by elderly Hong Kong Chinese: A senior technology acceptance model (STAM). Ergonomics.

[22] Venkatesh V., Morris M.G., Davis G.B., Davis F.D. (2003). User acceptance of information technology: Toward a unified view. MIS Q..

[23] Heerink M., Kröse B., Evers V., Wielinga B. (2010). Assessing acceptance of assistive social agent technology by older adults: The almere model. Int. J. Soc. Robot..

[24] Tsai T.H., Lin W.Y., Chang Y.S., Chang P.C., Lee M.Y. (2020). Technology anxiety and resistance to change behavioral study of a wearable cardiac warming system using an extended TAM for older adults. PLoS ONE.

[25] Abdulai A.F., Tiffere A.H., Adam F., Kabanunye M.M. (2021). COVID-19 information-related digital literacy among online health consumers in a low-income country. Int. J. Med. Inform..

[26] Kemp E., Trigg J., Beatty L., Christensen C., Dhillon H.M., Maeder A., Williams P.A.H., Koczwara B. (2021). Health literacy, digital health literacy and the implementation of digital health technologies in cancer care: The need for a strategic approach. Health Promot. J. Aust..

[27] Pechrapa K., Yodmai K., Kittipichai W., Charupoonpol P., Suksatan W. (2021). Health literacy among older adults during COVID-19 pandemic: A cross-sectional study in an urban community in Thailand. Ann. Geriatr. Med. Res..

[28] Erdfelder E.F., Buchner A. (1996). GPOWER: A general power analysis program. Behav. Res. Methods Instrum. Comput..

[29] Norman C.D., Skinner H.A. (2006). eHEALS: The eHealth literacy scale. J. Med. Int. Res..

[30] Venkatesh V., Bala H. (2008). Technology acceptance model 3 and a research agenda on interventions. Decis. Sci..

[31] Pett M.A., Lackey N.R., Sullivan J.J. (2003). Making Sense of Factor Analysis: The Use of Factor Analysis for Instrument Development in Health Care Research.

[32] Hong S., Malik M.L., Lee M.K. (2003). Testing configural, metric, scalar, and latent mean invariance across genders in sociotropy and autonomy using a non-Western sample. Educ. Psychol. Meas..

[33] Browne M.W., Cudeck R. (1992). Alternative ways of assessing model fit. Sociol. Methods Res..

[34] Hu L.T., Bentler P.M. (1999). Cutoff criteria for fit indexes in covariance structure analysis: Conventional criteria versus new alternatives. Struct. Equ. Model. A Multidiscip. J..

[35] Kim M.K., Kim J.H. (2006). A study on the prospect customers’ characteristics influencing intentions to use DMB service in Korea. Korean J. Broadcast. Telecommun. Stud..

[36] Hong S.H., Hwang M.H., Lee E.S. (2005). Latent means analysis of the career-barrier scale for Korean female adolescents. Korean J. Educ. Psychol..

[37] Cohen J. (1988). Statistical Power Analysis for the Behavioral Sciences.

[38] Cole D.A., Maxwell S.E., Arvey R., Salas E. (1993). Multivariate group comparisons of variable systems: MANOVA and structural equation modeling. Psychol. Bull..

[39] Geiser C., Burns G.L., Servera M. (2014). Testing for measurement invariance and latent mean differences across methods: Interesting incremental information from multitrait-multimethod studies. Front. Psychol..

[40] Eid M., Schneider C., Schwenkmezger P. (1999). Do you feel better or worse? The validity of perceived deviations of mood states from mood traits. Eur. J. Pers..

[41] Hancock G.R. (1997). Structural equation modeling methods of hypothesis testing of latent variable means. Meas. Eval. Couns. Dev..

[42] Levin-Zamir D., Bertschi I. (2018). Media health literacy, eHealth literacy, and the role of the social environment in context. Int. J. Environ. Res. Public Health.

[43] Neter E., Brainin E. (2012). eHealth literacy: Extending the digital divide to the realm of health information. J. Med. Internet Res..

[44] Do B.N., Tran T.V., Phan D.T., Nguyen H.C., Nguyen T.T.P., Nguyen H.C., Ha T.H., Dao H.K., Trinh M.V., Do T.V. (2020). Health literacy, eHealth literacy, adherence to infection prevention and control procedures, lifestyle changes, and suspected COVID-19 symptoms among health care workers during lockdown: Online survey. J. Med. Int. Res..

[45] Van Duong T., Chiu C.H., Lin C.Y., Chen Y.C., Wong T.C., Chang P.W., Yang S.H. (2021). E-healthy diet literacy scale and its relationship with behaviors and health outcomes in Taiwan. Health Promot. Int..

[46] Duong T.V., Nguyen T.T.P., Pham K.M., Nguyen K.T., Giap M.H., Tran T.D.X., Nguyen C.X., Yang S.H., Su C.T. (2019). Validation of the short-form health literacy questionnaire (HLS-SF12) and its determinants among people living in rural areas in Vietnam. Int. J. Environ. Res. Public Health.

[47] Van Hoa H., Giang H.T., Vu P.T., Van Tuyen D., Khue P.M. (2020). Factors associated with health literacy among the elderly people in Vietnam. BioMed Res. Int..

[48] Kayupova G., Turdaliyeva B., Tulebayev K., Van Duong T., Chang P.W., Zagulova D. (2017). Health literacy among visitors of district polyclinics in Almaty, Kazakhstan. Iran. J. Public Health.

[49] Davis F.D., Bagozzi R.P., Warshaw P.R. (1989). User acceptance of computer technology: A comparison of two theoretical models. Manag. Sci..

[50] Bol N., Helberger N., Weert J.C. (2018). Differences in mobile health app use: A source of new digital inequalities?. Inf. Soc..

[51] Murray T.S., Hagey J., Willms D., Shillington R., Desjardins R. (2008). Health Literacy in Canada: A Healthy Understanding.

[52] Sun H., Zhang P. (2006). The role of moderating factors in user technology acceptance. Int. J. Hum.-Comput. Stud..

[53] Lawton M.P., Nahemow L. (1973). Ecology and the Aging Process.

[54] Kamin S.T., Lang F.R., Kamber T. (2016). Social Contexts of Technology Use in Old Age.

